# Cloning, Expression, Purification, and Characterization of a Novel β-Galactosidase/α-L-Arabinopyranosidase from *Paenibacillus polymyxa* KF-1

**DOI:** 10.3390/molecules28227464

**Published:** 2023-11-07

**Authors:** Jing Cui, Yibing Wang, Andong Zhou, Shuhui He, Zihan Mao, Ting Cao, Nan Wang, Ye Yuan

**Affiliations:** 1 Institute of Innovation Science & Technology, Central Laboratory, Changchun Normal University, Changchun 130031, China; cuijing@ccsfu.edu.cn; 2 Engineering Research Center of Glycoconjugates Ministry of Education, Jilin Provincial Key Laboratory of Chemistry and Biology of Changbai Mountain Natural Drugs, School of Life Sciences, Northeast Normal University, Changchun 130024, China; wangyb302@nenu.edu.cn (Y.W.); adzhou@nenu.edu.cn (A.Z.); hesh207@nenu.edu.cn (S.H.); maozh902@nenu.edu.cn (Z.M.); wangn179@nenu.edu.cn (N.W.)

**Keywords:** β-galactosidase, α-L-arabinopyranosidase, bifunctional enzyme, GH42, *Paenibacillus polymyxa* KF-1

## Abstract

Glycosidases are essential for the industrial production of functional oligosaccharides and many biotech applications. A novel β-galactosidase/α-L-arabinopyranosidase (PpBGal42A) of the glycoside hydrolase family 42 (GH42) from *Paenibacillus polymyxa* KF-1 was identified and functionally characterized. Using *p*NPG as a substrate, the recombinant PpBGal42A (77.16 kD) was shown to have an optimal temperature and pH of 30 °C and 6.0. Using *p*NPαArap as a substrate, the optimal temperature and pH were 40 °C and 7.0. PpBGal42A has good temperature and pH stability. Furthermore, Na^+^, K^+^, Li^+^, and Ca^2+^ (5 mmol/L) enhanced the enzymatic activity, whereas Mn^2+^, Cu^2+^, Zn^2+^, and Hg^2+^ significantly reduced the enzymatic activity. PpBGal42A hydrolyzed *p*NP-β-D-galactoside and *p*NP-α-L-arabinopyranoside. PpBGal42A liberated galactose from β-1,3/4/6-galactobiose and galactan. PpBGal42A hydrolyzed arabinopyranose at C20 of ginsenoside Rb2, but could not cleave arabinofuranose at C20 of ginsenoside Rc. Meanwhile, the molecular docking results revealed that PpBGal42A efficiently recognized and catalyzed lactose. PpBGal42A hydrolyzes lactose to galactose and glucose. PpBGal42A exhibits significant degradative activity towards citrus pectin when combined with pectinase. Our findings suggest that PpBGal42A is a novel bifunctional enzyme that is active as a β-galactosidase and α-L-arabinopyranosidase. This study expands on the diversity of bifunctional enzymes and provides a potentially effective tool for the food industry.

## 1. Introduction

β-Galactosidases (EC 3.2.1.23), which hydrolyze β-D-galactose residues at the non-reducing end of sugar conjugates, are used in dairy processing [1], oligogalactose synthesis [2], enzyme replacement treatment [3,4], and genetic screening [5]. The physical and biological properties of naturally occurring plant cell wall polysaccharides and their corresponding oligosaccharides are of great interest, and many have been used as functional food ingredients [6]. β-galactosidase catalyzes the hydrolysis of lactose into glucose and galactose, and also takes part in the transgalactosylation reaction that produces galato-oligosaccharide (GOS) (e.g., Gal (β1→3) Gal (β1→4) Gal (β1→6)) [7,8]. Lactose is the most common disaccharide found in mammalian milk. Individuals with lactose intolerance are congenitally unable to decompose lactose and experience various symptoms, such as abdominal pain and diarrhea when consuming dairy products. Approximately 70% of the world population and more than 90% of East Asians suffer from lactose intolerance [9]. Therefore, β-galactosidase is widely used to produce low-lactose milk in people with lactose intolerance. β-Galactosidase can also degrade galactan or arabinogalactan to oligosaccharides, which are considered prebiotics [10]. α-L-arabinopyranosidases (EC 3.2.1.-) are a class of arabinoglycosidases that break down the non-reducing end of α-L-arabinopyranoside bonds in sugars that contain L-arabinose. α-L-arabinopyranose is a component of many plant polysaccharides and arabinoglycans, and α-L-arabinopyranosidase has potential applications in the biotransformation of these substances [11,12]. Ginsenoside Rb2, which is the main components of ginseng (the root of *Panax ginseng* C.A. Meyer, Araliaceae), is an important medicinal herb in Asia. Ginsenoside Rd has shown an inhibitory effect on carrageenan-induced inflammation, a promotive effect on neural stem cells, and a wound-healing effect [13]. Ginsenoside Rd is structurally similar to Rb2, but lacks one outer glycoside moiety at position C20. Therefore, Rb2 can be transformed into Rd by the cleavage of the outer arabinopyranose by α-L-arabinopyranosidase.

Based on their amino acid sequences and reaction mechanism, β-galactosidases belong to glycoside hydrolase (GH) families 1, 2, 35, 39, 42, 59, 147, and 165 and have been functionally characterized from different sources, such as plants, animals, bacteria, and fungi, etc. [14,15,16]. α-L-arabinopyranosidase was initially purified from *B. breve* K-110 in *Bifidobacterium* species [17]. Until now, there were few studies on α-L-arabinopyranosidase, which have been reported to belong to the families GH 2 and 42 [11,12,13,18,19]. Bifunctional enzymes are more efficient, economical, and suitable for industrial applications. Until now, the majority of GH42 enzymes are β-galactosidases without reported α-L-arabinopyranosidase activity, and few GH42 bifunctional enzymes (β-galactosidase/α-L-arabinopyranosidase) have been reported from *Bacillus* sp. KW1 [19] and *Bifidobacterium longum* H-1 [12]. Therefore, we must expand the sources of bifunctional enzymes and promote the research and application of GH42 enzymes in biotechnology.

*Paenibacillus polymyxa* is a class of aerobic (or partially anaerobic) bacteria that is widely distributed in nature. This bacterium can grow well in relatively harsh environments with hyperosmolarity, high acidity, high alkalinity, and high or low temperatures [20]. Moreover, it produces a variety of extracellular hydrolases in the defense against pathogenic bacteria and is widely used in various industrial applications. The enzymes currently extracted from *Paenibacillus polymyxa* include the pectinases of the polysaccharide lyase family 9 (PL9) and PL10 families, and the glucanases of the GH5 family [21,22]. However, none of the β-galactosidases/α-L-arabinopyranosidases from *Paenibacillus polymyxa* have been characterized. In the present study, a novel β-galactosidase/α-L-arabinopyranosidase gene (PpBGal42A) of GH42 from *Paenibacillus polymyxa* KF-1 was cloned and expressed in *Escherichia coli*, and the recombinant PpBGal42A was purified. The enzymatic properties of PpBGal42A were comparatively characterized in detail to elucidate their feasibilities for application in polysaccharide degradation and in other biotech applications.

## 2. Results

### 2.1. Gene Cloning and Analysis of PpBGal42A

**Figure 1:**
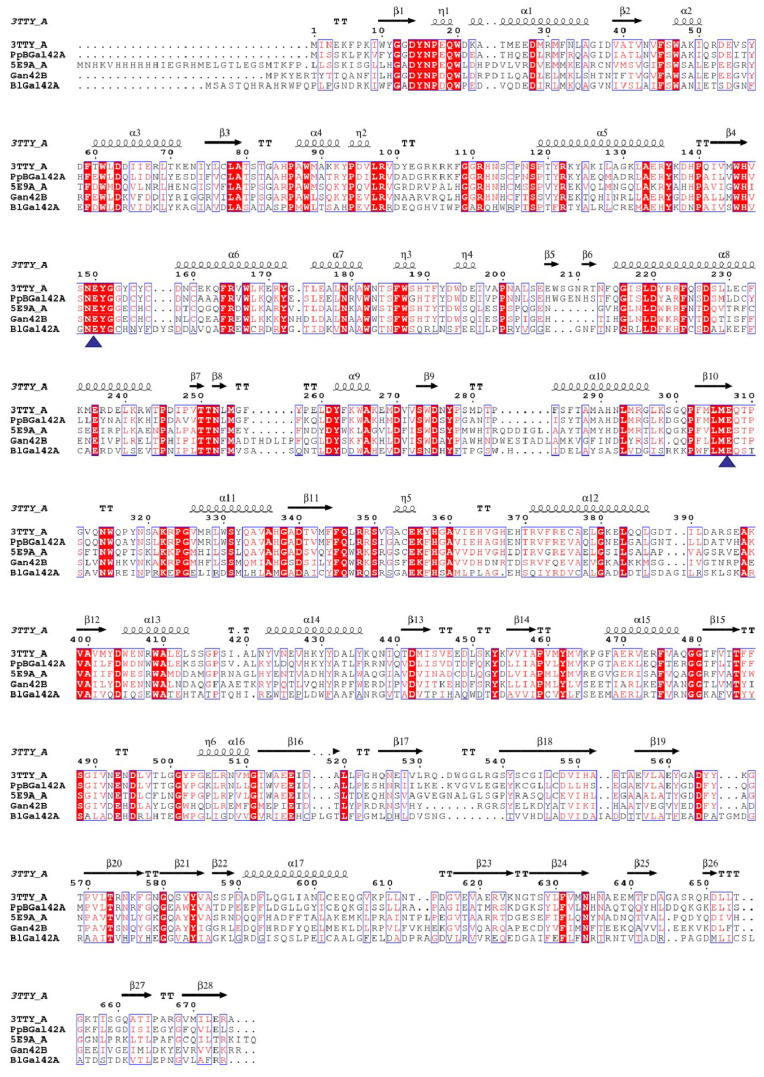
Comparison of amino acid sequences of enzymes from the GH42 family. PpBGal42A is aligned with a β-galactosidase from *Bacillus circulans* sp. *Alkalophilus* (PDB:3TTS_A) [23], *Rahnella* sp. R3 (PDB:5E9A) [24], Gan42B from *Geobacillus stearothermophilus* (PDB:4OIF) [25], and BlGal42A from *Bifidobacterium animalis* subsp. *lactis* Bl-04 (PDB:4UNI) [26]. The two Glu amino acids are denoted with solid triangles.

The coding cDNA for the gene PpBGal42A from *P. polymyxa* KF-1 was 2028 bp, as stated in the NCBI (GenBank accession number: WP040101590.1). This cDNA encoded 675 amino acid residues with a pI of 5.23. The theoretical molecular mass of PpBGal42A is 77.16 kDa. We compared PpBGal42A with previously published enzymes (Figure 1) in the GH42 family. PpBGal42A showed the greatest similarity (66% identity) with a β-galactosidase from *Bacillus circulans* sp. *Alkalophilus* [23], followed by a 43% similarity to a cold-amplified beta-galactosidase from *Rahnella* sp. R3 [24], a 38% similarity to Gan42B from *Geobacillus stearothermophilus* [25], and a 32% similarity to BlGal42A from *Bifidobacterium animalis* subsp. *lactis* Bl-04 [26]. PpBGal42A has relatively low identity with other-galactosidases. Furthermore, two amino acids, Glu150 and Glu307, were predicted to be the catalytic acid/base and nucleophile, respectively [27].

**Figure 2:**
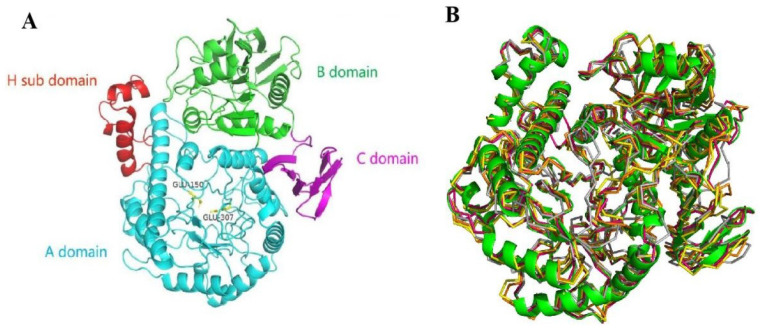
The three-dimensional structure of the PpBGal42A monomer. (**A**) Ribbon diagram showing the 3D structure of PpBGal42A. The A, B, and C domains are shown in blue (with the H subdomain in red), green, and purple. Catalytic residues Glu150 and Glu307 are labeled and shown as yellow sticks. (**B**) Structural superposition of PpBGal42A (green) with a β-Gal (PDB:3TTS, red) [23], R-β-Gal (PDB:5E9A, orange) [24], Gan42B (PDB:4OIF, yellow) [25], and BlGal42A (PDB:4UNI, gray) [26]. The figure was prepared using Pymol software 2.5.2.

The 3D-modeled structure of PpBGal42A was constructed using SWISS-MODEL and the structure of the galactosidase from *Bacillus circulans* sp. *Alkalophilus* (PDB accession number: 3TTS, identity = 65.82%) as a template [23]. The GMQE and QMEAN values for the homology model were 0.91 and 0.89, respectively, indicating a good model quality and high reliability. The structure of the resulting PpBGal42A monomer is shown in Figure 2A, which shows three distinct domains: domain A (residues 1–396), domain B (397–608), and domain C (609–673). The homologous modeling results showed that PpBGal42A preserved the catalytic sites (Asn149, Glu150, Met306, and Glu307). A comparison of PpBGal42A with four other homologous GH42-galactosidase structures reported to date indicated a relatively high structural similarity (Figure 2B). All proteins were composed of three relatively similar domains per monomer; however, although domain A seemed to be very similar between these structures, domains B and C seemed to vary more significantly in their general conformation and in some local loop structures (Figure 2B).

### 2.2. Expression and Purification of Recombinant PpBGal42A

**Figure 3:**
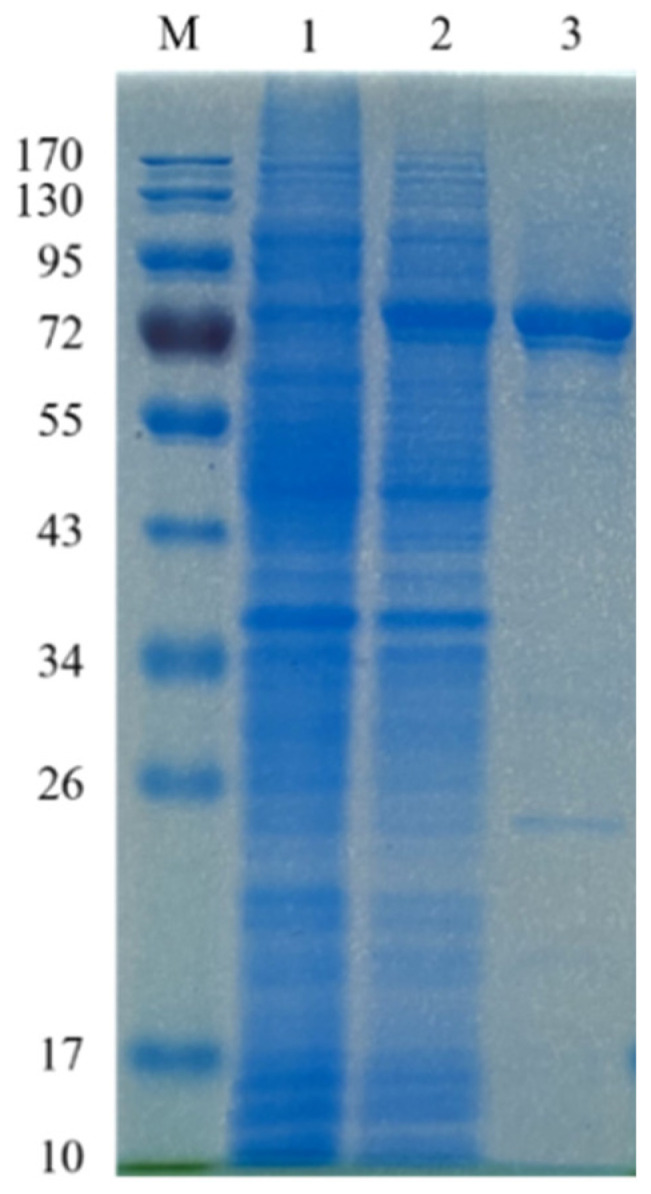
Mw of PpBGal42A determined by SDS-PAGE: M, Mw marker (PageRuler Prestained Protein Ladder, Thermo Scientific, Waltham, MA, USA). (1) Culture lysate before IPTG induction; (2) culture lysate after IPTG induction; (3) recombinant enzyme PpBGal42A purified from Ni-NTA agarose column.

PpBGal42A was cloned into the pET-28a (+) vector and overexpressed in *E. coli* BL21 (DE3). PpBGal42A was purified using Ni-NTA chromatography and obtained with 30.2 mg from 500 mL of LB medium. The enzyme showed a specific activity of 13.19 U/mg against *p*NPG. PpBGal42A has a molecular weight of approximately 77 kDa, as assessed by SDS-PAGE (Figure 3). This value is consistent with the predicted molecular weight.

### 2.3. Characterization of Recombinant PpBGal42A

**Figure 4:**
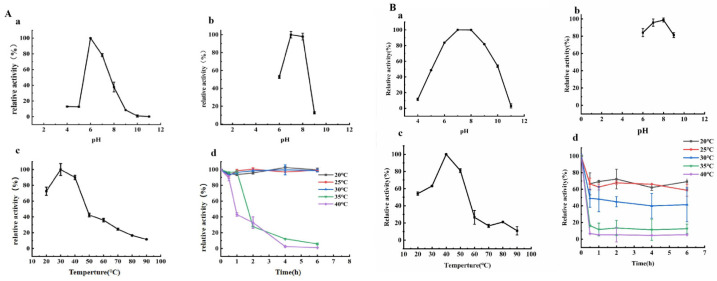
Effect of pH and temperature on activity and stability of PpBGal42A. (**A**) Using *p*NPG as substrate. (**A**-**a**) Optimal pH. (**A**-**b**) pH stability. (**A**-**c**) Optimal temperature. (**A**-**d**) Temperature stability. (**B**) Using *p*NPαArap as substrate. (**B**-**a**) Optimal pH. (**B**-**b**) pH stability. (**B**-**c**) Optimal temperature. (**B**-**d**) Temperature stability. Relative activity was calculated using the maximum activity as 100%. Results are presented as the mean ± standard deviation (*n* = 3).

Using *p*NPG as a substrate, the effect of pH on PpBGal42A activity was investigated in the range pH4.0–11.0. With optimal activity at pH 6.0, the enzyme exhibited good stability at pH 7.0–8.0, and retained >95% activity after 12 h of incubation at 4 °C. The optimal temperature for PpBGal42A was found to be 30 °C, and the PpBGal42A activity decreased sharply when the temperature reached 35 °C. The protein was incubated for 6 h at 20 °C to 40 °C, and maintained >90% activity at temperatures 20–30 °C (Figure 4A). Using *p*NPαArap as a substrate, the optimal activity was at pH 7.0, and the enzyme exhibited good stability at pH 6.0–9.0 and retained >80% activity. The optimal temperature for PpBGal42A was found to be 40 °C, and maintained >60% activity at temperatures 20–25 °C (Figure 4B).

As shown in Table 1, the effects of metal ions and chemical reagents on the activity of PpBGal42A were investigated. PpBGal42A was weakly activated by Na^+^, K^+^, Li^+^, and Ca^2+^ (5 mmol/L). Meanwhile, PpBGal42A was completely inhibited by Mn^2+^, Cu^2+^, Zn^2+^, and Hg^2+^ (5 mmol/L), and was significantly inhibited by Fe^2+^ and Ni^2+^ (5 mmol/L) (Table 1). The Michaelis–Menten parameters for PpBGal42A with *p*NPG as a substrate were determined as a *K*m of 1.1 ± 0.2 g/L and *V*m of 232.6 ± 9.8 μmol/min/mg.

### 2.4. Substrate Specificity of PpBGal42A

**Figure 5:**
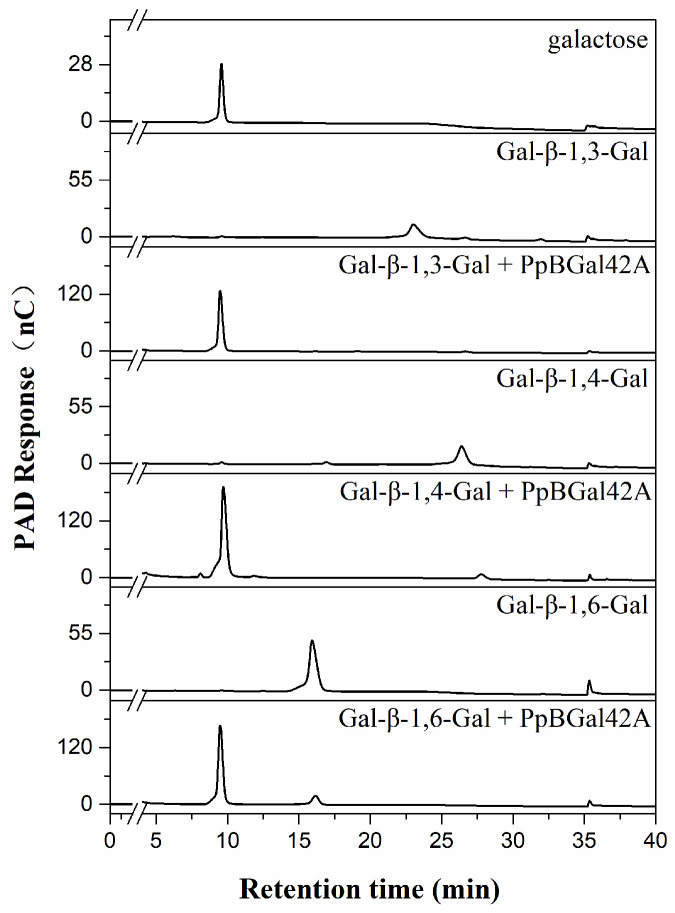
HPAEC-PAD analysis of hydrolysates of PpBGal42A on β-1,3/4/6-linked galactobiose. D-galactoses were used as standards.

The substrate specificity of PpBGal42A was evaluated using *p*NP-glycosidase and several disaccharides and polysaccharides. Among the 12 *p*NP glycosides, PpBGal42A displayed significant activity towards *p*NPG (100%), moderate activity towards *p*NP-α-L-arabinopyranoside (56.7%), and no activity towards the other substrates (Table 2). Disaccharides with different linkages were used to identify the glycosidic bond specificity. With the release of galactose, PpBGal42A completely hydrolyzed β-1,3-galactobiose, hydrolyzed most of β-1,4-galactobiose, andβ-1,6-galactobiose (Figure 5). PpBGal42A hydrolyzed arabinopyranose at C20 of ginsenoside Rb2 and converted it to Rd. PpBGal42A could not cleave arabinofuranose at C20 of ginsenoside Rc (Figure S1).

**Figure 6:**
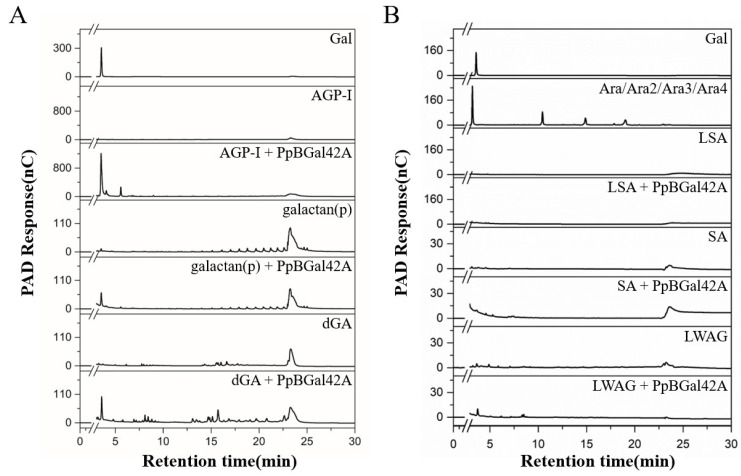
HPAEC-PAD analysis of degradation products of different polysaccharides by PpBGal42A. (**A**) AGP-I, galactan (p), dGA; (**B**) LSA, SA, and LWAG. D-galactose and L-arabinose were used as standards.

Additionally, the substrate specificity of PpBGal42A was assayed using polysaccharides. Our results showed that recombinant PpBGal42A released galactose from AGP-I (prepared in our laboratory), potato galactan (galactan (p)) (main-chain glycosidic linkage: β-1,4,Gal:Ara:Rha:GalA = 87:3:4:6), and dGA (prepared in our laboratory) (Figure 6A). However, it did not release arabinose from linear arabinosaccharides (LSA; more than 95% purity, Ara:Gal:Rha:GalA = 85.2:7.6:1.5:5.7), arabinosaccharides (SA; purity approximately 95%, Ara:Gal:Rha:GalA:other sugars = 69:18.7:1.4:10.2:0.7), and LWAG (>95% purity, Gal:Ara:other sugars = 81:14:5) (Figure 6B). This indicated that PpBGal42A has exo-β-1,3/4/6-galactanase activity.

### 2.5. Hydrolysis of Lactose

**Figure 7:**
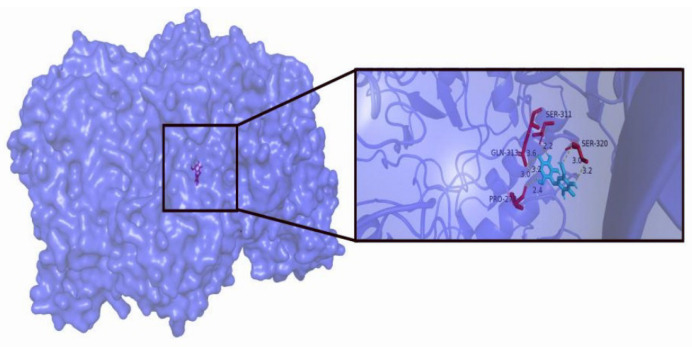
Molecular docking of PpBGal42A with lactose. Overall structure and substrate binding pocket analysis of PpBGal42A. Lactose is blue, AA residues (Pro278, Gln313, Ser311, and Ser320) are red.

**Figure 8:**
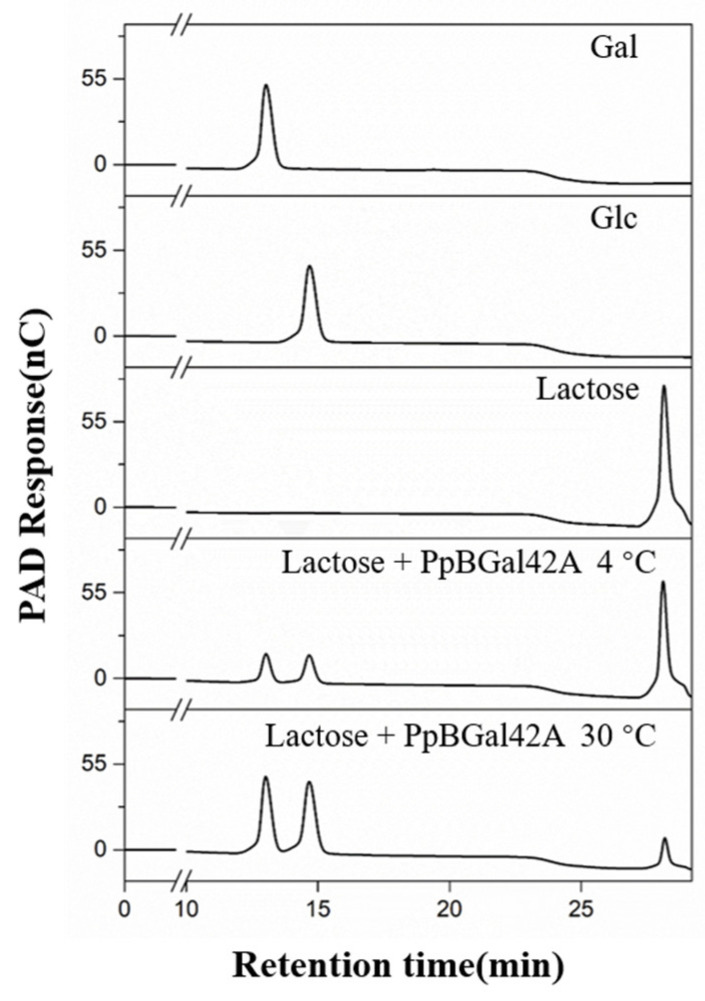
HPAEC-PAD analysis of lactose hydrolysates by PpBGal42A. Standards are Gal and Glc.

Lactose was docked into the substrate-binding pocket of PpBGal42A to generate a binding mode. The docking results revealed that lactose bound to the active site pocket of PpBGal42A (Figure 7). Substrate–enzyme interaction analyses were performed to determine the substrate recognition mechanisms. Four AA residues (Pro278, Gln313, Ser311, and Ser320) formed seven hydrogen bonds with lactose. These results show that PpBGal42A has a strong lactose-binding ability, which is beneficial for substrate hydrolysis. The binding energy between PpBGal42A and lactose was −8.2 kcal/mol, which was lower than that of the binding energy (−7.7 kcal/mol) between B. circulans (PDB:3TTS) and lactose. The hydrolysis rate of lactose with PpBGal42A reached about 22.6%, and 82.0% at 4 °C, and 30 °C after 24 h incubation, respectively (Figure 8).

### 2.6. PpBGal42A and Pectinase Display Synergistic Activity in Degrading Citrus Pectin

When citrus pectin was incubated with pectinase for 24 h, we observed that ~579 μg of reducing sugar was released. Meanwhile, the incubation of citrus pectin with PpBGal42A for 24 h released ~3.5 μg of reducing sugar. However, when both enzymes were combined, 642 μg of reducing sugar was released from citrus pectin, which is a 1.1-fold increase compared to their individual use. This suggested a relatively modest synergistic effect (Table 3).

## 3. Discussion

*Paenibacillus polymyxa* is a potentially important biotechnological agent [20], because it efficiently produces an array of compounds that are useful in industrial processes. In this study, we cloned and expressed the β-galactosidase/α-L-arabino-pyranosidase gene PpBGal42A from *Paenibacillus polymyxa* KF-1 with an expression level of up to 60.4 mg/L and specific activity towards *p*NPG (13.19 U/mg). SDS-PAGE showed that purified PpBGal42A appeared as a single band with a relative molecular mass of ~77 kDa.

Using *p*NPG as substrate, the optimal pH for PpBGal42A activity was pH 6.0, and ~95% activity was maintained in the pH range of 7–8. β-galactosidase. A pH between 6–7.5 is suitable for hydrolyzing the lactose present in milk and sweet whey [4]. The optimal temperature for PpBGal42A was observed to be 30 °C, with ~90% activity being maintained at temperatures ranging from 20 °C to 30 °C. Using *p*NPαArap as substrate, the optimal temperature was 40 °C, the optimal activity was at pH 7.0, and the enzyme exhibited good stability at pH 6.0–9.0, and retained >80% activity. PpBGal42A exhibited a relatively higher temperature and pH stability. The pH and temperature ranges support the idea that PpBGal42A is a potentially useful bioindustrial tool. Enzymes generally face unfavorable reaction conditions when used in industrial bioprocesses, and thus must have the ability to tolerate various reaction conditions. The stability of β-galactosidase was investigated in the presence of various metal ions and surfactants. Mono- and divalent cations affect β-galactosidase activity [28]. K^+^ and Na^+^ enhance the activity of Gal3149 from *Bacillus velezensis* SW5, while Zn^2+^ and Cu^2+^ strongly or completely inhibit its activity [29]. The divalent cations Mg^2+^, Ca^2+^, and Zn^2+^ were found to enhance the catalytic activity of BgaC, whereas Cu^2+^ and Mn^2+^ were inhibitory [30]. In this study, PpBGal42A was slightly activated by Na^+^, K^+^, Li^+^, and Ca^2+^ (5 mmol/L). PpBGal42A was significantly or completely inhibited by 5 mM Mn^2+^, Cu^2+^, Zn^2+^, Hg^2+^, Fe^2+^, or Ni^2+^.

The GH42 family of galactosidases hydrolyzes different types of galactoside bonds. For instance, BlGal42A displays a preference for undecorated β-1,3 and β-1,6 linked galactosides [31]. Bga42A prefers the β-1,3-galactosidic linkage from human milk and other β-1,3 and β-1,6-galactosides with glucose or galactose situated at subsite +1 [32]. Β-Gal II is highly active towards β-1,4-galactosides and galacto-oligosaccharides containing (β-1-4) linkages and shows weak trans-glycosylation activity at low substrate concentrations [33]. Bga42B very efficiently hydrolyzes 4-galactosyllactose (Galβ1-4Galβ1-4Glc), as well as 4-galactobiose (Galβ1-4Gal) and 4-galactotriose (Galβ1-4Galβ1-4Gal) [32]. In this study, PpBGal42A not only hydrolyzed β-1,3-galactosides, but also hydrolyzed β-1,4/6-galactosides, and has high exo-β-1,3/4/6-galactanase activity. Enzymes belonging to GH42 are ubiquitous in microbes and usually have two types of enzymatic activity (galactosidase and L-arabinopyranosidase). Comparison with other bifunctional enzymes (β-galactosidase/α-L-arabinopyranosidase) from various microorganisms, as shown in Table 4, the GH42 bifunctional β-galactosidase/α-L-arabinopyranosidase from *B. longum* was reported to degrade *p*NPG and *p*NPA [12], whereas BgaA from *Clostridium cellulovorans* [34] and Gan42B from *Geobacillus stearothermophilus* [35] were reported to degrade *p*NPG, *p*NPA, and *p*NPF. The GH42 enzymes from *Bacillus* sp. KW1 [18] hydrolyze *p*NPG, *p*NPA, *p*NPF, and *o*NPG. PpBGal42A and Gan42B [35] have been reported to exhibit L-arabinopyranosidase activity towards plant glycosides, including the degraded ginsenoside Rb2. However, no bifunctional enzyme has been reported to degrade β-1,3/4/6-galactan. Thus, it is important to identify other bifunctional enzymes that may provide potentially effective tools for biotechnological applications. In this study, PpBGal42A hydrolyzed not only *p*NP-β-D-galactoside, β-1,3/4/6-galactobiose, and galactan, but also *p*NP-α-L-arabinopyranoside. We identified PpBGal42A as a bifunctional enzyme with exo-β-1,3/4/6-galactanase and -L-arabinopyranosidase activities. The bifunctional enzyme may reduce the cost compared to the combination of these enzymes and may catalyze the hydrolysis of polysaccharides better than a combination of enzymes.

We also studied the ability of PpBGal42A to degrade lactose. Lactose is partially degraded to galactose and glucose at 30 °C or 4 °C and may be completely degraded when the amount of PpBGal42A is increased or when the reaction time is prolonged. This feature enables the use of this enzyme for the removal of lactose from dairy products. The hydrolysis of lactose in milk can be performed chemically or enzymatically. β-Galactosidase is widely used in the production of lactose-free dairy products because it avoids the production of byproducts and does not alter the physicochemical properties of milk [16,36]. These products are intended for consumption by lactose-intolerant patients whose digestive systems are deficient in β-galactosidase [37]. In addition, β-galactosidase is used to prepare ice cream and condensed milk to avoid lactose crystallization and enhance the sweetness and creaminess of these products [38].

Our in-depth analysis of the hydrolytic characteristics of PpBGal42A supports its use in specific instances. For example, PpBGal42A exhibits significant degradative activity towards citrus pectin when combined with pectinase. Our findings underscore the significant benefits of this pair of enzymes when delineating structure–function relationships in polysaccharides and their biological functions. We expect that PpBGal42A will be an excellent candidate for the identification of fruit juice pectin.

## 4. Materials and Methods

### 4.1. Strains and Reagents

*P. polymyxa* KF-1 was isolated, identified in our laboratory, and preserved at the China General Microbiological Culture Collection Center (collection number: CCTCC AB 2018146). *Escherichia coli* BL21 (DE3) pET-28a (+) (Novagen, Madison, WI, USA) was used as the expression vector. *p*NP-β-D-galactopyranoside (*p*NPG), *p*NP-α-D-galactopyranoside (*p*NPαGal), *p*NP-α-L-arabinofuranoside (*p*NPαAraf), *p*NP-α-L-arabinopyranoside (*p*NPαArap), *p*NP-α-D-glucopyranoside (*p*NPαGlc), *p*NP-β-D-glucopyranoside (*p*NPβGlc), *p*NP-α-D-mannopyranoside (*p*NPαMan), *p*NP-β-D-mannopyranoside (*p*NPβMan), *p*NP-α-D-xylopyranoside (*p*NPαXyl), *p*NP-β-D-xylopyranoside (*p*NPβXyl), *p*NP-α-L-fucopyranoside (*p*NPαFuc), and *p*NP-α-L-rhamnoside (*p*NPαRha) were purchased from Sigma (St. Louis, MO, USA). The ginsenosides Rb2 and Rc were prepared in our laboratory. Disaccharides (galactose-β-(1→3)-galactose, galactose-β-(1→4)-galactose, galactose-β-(1→6)-galactose), potato galactan (β-1,4-galactan), larch wood arabinogalactian (LWAG), sugar beet arabinan (product code: P-ARAB) (SA), and linear sugar beet arabinan (product code: P-LARB) (LSA) were purchased from Megazyme International Ireland Ltd. (Wicklow, Ireland). AGP-I(β-1,3-galactan) was prepared by Smith degradation of larch arabinogalactan, followed by oxidation with periodate [39]. dGA (β-1,6-galactan) was prepared by partial acid hydrolysis of gum arabic [40]. Citrus pectin (Solarbio, Galactronic Acid ≥ 58.0%) and pectinase (1.12 U/mg) were from Sigma (St. Louis, MO, USA). All other chemicals and reagents were of analytical grade.

### 4.2. Sequence Analysis and Modeling of PpBGal42A

Signal peptides were predicted using SignalP 5.0. Protein sequences were obtained from the National Center for Biotechnology Information (NCBI). Sequence alignment was performed using Clustal Omega (http://www.ebi.ac.uk/Tools/msa/clustalo/, accessed on 30 March 2023) [41]. The three-dimensional (3-D) structure of PpBGal42A was predicted using the SWISS-MODEL (https://swissmodel.expasy.org accessed on 30 March 2023) [31]. Secondary structural elements and key catalytic residues based on alignments with the most related-galactosidase entries from the RCSB Protein Data Bank (PDB) (https://www.wwpdb.org accessed on 30 March 2023) were depicted using the online tool ESPript version 3 [42]. Molecular graphics were visualized and presented using the PyMOL molecular graphics system (version 2.3.0, DeLano Scientific LLC, Palo Alto, CA, USA).

### 4.3. Construction of Plasmids and Strains

Total DNA was extracted from *P. polymyxa* KF-1 using a DNA Extraction Kit. Two primers, forward (5′-GACTGGTGGACAGCAAATGGGTCGCGGATCCA-TGATAAGCAGCAAACTTCC-3′) and reverse (5′-GATCTCAGTGGTGGTGGTGGTGGTGCTCGAGTTAGGATAGCTCCAGCACTT-3′) that contain restriction sites for *BamHI* and *XhoI* (restriction sites underlined), respectively, were designed based on the gene. PCR was performed using 2 × Taq PCR Green Mix with the following protocol: 94 °C for 3 min, 30 cycles at 94 °C for 30 s, 56 °C for 30 s, 72 °C for 2 min, and finally at 72 °C for 5 min. The PCR product and pET-28a (+) were digested with BamHI and XhoI and ligated with pET-28a (+) to generate the recombinant plasmid pET-28a-PpBGal42A. All enzymes were obtained from New England Biolabs (Beverly, MA, USA). Restriction enzyme digestion, ligations, and transformations were performed according to the supplier’s recommendations.

### 4.4. Expression and Purification of Recombinant PpBGal42A

By heat shock, pET-28a-PpBGal42A was transformed into *E. coli* BL21 (DE3). The positive transformants were verified by DNA sequencing (Sangon Biotech, Shanghai, China). The *E. coli* transformants were grown in LB medium supplemented with 50 μg/mL kanamycin. When the optical density at 600 nm reached 0.6–0.8, IPTG (final concentration of 0.5 mM) was added, and the incubation was continued at 16 °C for 20 h, followed by centrifugation at 8000 rpm for 10 min to collect cells and ultrasonically disrupt them. The supernatant was obtained by centrifugation at 13,000 rpm for 30 min and purified by Ni-NTA affinity chromatography. The recombinant enzyme was purified on a Ni+ Sepharose Fast Flow column (GE Healthcare, Chicago, IL, USA). A flow pump was used to maintain the binding rate at 1 mL/min. After binding, the impure protein was eluted with 20 mM imidazole, 0.1 M NaCl, and 20 mM phosphate buffer (pH 7.0). The target protein was eluted with a high concentration of imidazole (300 mM) in 20 mM phosphate buffer (pH 7.0). Purified PpBGal42A was analyzed by sodium dodecyl sulfate-polyacrylamide gel electrophoresis (SDS-PAGE) on a 10% separating gel [43].

### 4.5. Characterization of Recombinant PpBGal42A

The optimal pH, pH stability, optimal temperature, and temperature stability for PpBGal42A were determined using 5 mM *p*NPG and *p*NPαArap as substrate, respectively. The optimal pH for PpBGal42A was determined at 30 °C using *p*NPG as substrate in a 20 mM buffer (Britton–Robinson’s universal pH buffer) for pH 4 to pH 11 [25], and at 40 °C using *p*NPαArap as substrate. The pH stability was investigated under standard assay conditions following incubation of the purified enzyme for 12 h at 4 °C in the buffer without substrate, and by monitoring the reaction at 405 nm and comparing results to a non-incubated sample. The optimal temperature of PpBGal42A was determined at temperatures ranging from 20–90 °C. The temperature stability of PpBGal42A was examined by incubating the purified enzyme for 6 h at different temperatures between 20–40 °C.

The effects of metal ions and chemicals on PpBGal42A were also assessed. The additives were diluted with NaAc-HAc buffer (pH 6.0) to final concentrations of 5 mM and 50 mM, and residual activity was measured under standard conditions (30 °C, 5 min). Kinetic parameters for PpBGal42A were determined using *p*NPG as substrate in 20 mM NaAc-HAc buffer (pH 6.0), 0.1 mM to 10 mM *p*NPG at 30 °C for 5 min. *K*m and *V*max values were calculated using GraphPad Prism V5 software.

### 4.6. Substrate Specificity

To investigate substrate specificity, purified PpBGal42A was used to hydrolyze 12 chromogenic substrates: *p*NPG, *p*NPαGal, *p*NPαAraf, *p*NPαArap, *p*NPαGlc, *p*NPβGlc, *p*NPαMan, *p*NPβMan, *p*NPαXyl, *p*NPβXyl, *p*NPαFuc, and *p*NPαRha. Hydrolytic activity towards *p*NP-glycosidase was determined at 30 °C in 20 mM NaAc-HAc buffer (pH 6.0) with the molar ratio of enzyme to substrate as 1:1000. After incubation for 5 min, the liberated *p*NPs were measured using a spectrophotometer and monitored at 405 nm.

To explore the hydrolytic activity of PpBGal42A, different disaccharides (galactose-β-(1→3)-galactose, galactose-β-(1→4)-galactose, and galactose-β-(1→6)-galactose) were selected. The reaction mixture (200) consisted of 5 μg purified PpBGal42A and 0.5 mg substrate in 20 mM NaAc-HAc buffer (pH 6.0) at 30 °C for 24 h. The released products were detected using high-performance anion-exchange chromatography (HPAEC) with a CarboPac PA-210 column (4 mm × 150 mm) attached to a Dionex ICS-5000 Plus ion chromatographic system. The protocol was as follows: 0–20 min, 10 mM NaOH elution; 20–40 min, 10 mM NaOH, 0 mm–40 mM NaAc linear gradient elution; 40–45 min, 100 mM NaOH, 300 mM NaAc elution; 45–50 min, 200 mM NaOH elution.

Ginsenosides Rb2 and Rc with different arabinose configurations at C20 were evaluated as substrates. The hydrolyzing capacity of PpBGal42A (10 μg/mL) was determined using 2.0 mg/mL of Rb2 and Rc as substrates in 20 mM NaAc-HAc buffer (pH 6.0) at 30 °C. The reaction solution containing ginsenosides was extracted using an equal volume of water-saturated n-butanol. After centrifugation, the n-butanol fraction was examined by TLC using 60F254 silica gel plates (Merck, Darmstadt, Germany) with n-butanol: ethyl acetate:water (4:4:1, *v*/*v*) as the solvent. TLC plates were sprayed with 10% (*v*/*v*) H_2_SO_4_, followed by heating at 110 °C for 3 min to visualize ginsenoside spots, which were identified by comparison with a standard.

Different polysaccharides (AGP-I (β-1,3-galactan), potato galactan (β-1,4-galactan), dGA (β-1,6-galactan), larch wood arabinogalactian (LWAG), arabinosaccharides (SA), and linear arabinosaccharides (LSA)) were used to evaluate the hydrolytic activities of PpBGal42A. After 24 h of reaction at 30 °C, samples were tested for molecular weight changes using TSK-G3000. The released products were detected by HPAEC using a CarboPac PA-200 column (3 × 250 mm) attached to a Dionex ICS-5000 Plus ion chromatographic system, using the protocol: 0–10 min, 50 mM NaOH; 10–30 min linear gradient of 0–100 mM NaAc in 5 mM NaOH; 30–40 min, 500 mM NaAc in 200 mM NaOH [44].

### 4.7. Hydrolysis of Lactose

Lactose was hydrolyzed at 4 °C and 30 °C for 24 h, and the products were detected by HPAEC using a CarboPac PA-20 column (3 × 150 mm), using the following protocol: 0–20 min, 2 mM NaOH elution; 20–35 min, 2 mM NaOH, 0 mM–200 mM NaAc linear gradient elution; 35–45 min, 200 mM NaOH elution. The sampling volume was 25 μL, and the flow rate was 0.4 mL/min from 0–45 min. The column temperature was 30 °C.

### 4.8. Synergistic Action of PpBGal42A with Pectinase with Citrus Pectin

Degradation of citrus pectin was performed by incubating 1 mg/mL citrus pectin with PpBGal42A or pectinase (or both) in 20 mM NaAc-HAc buffer (pH 6.0) at 30 °C for 24 h. The amount of reducing sugar released was measured using the DNS method [45], and comparing it to a standard curve generated using galactose.

## 5. Conclusions

In this study, we cloned and characterized the bifunctional enzyme (β-galactosidase/α-L-arabinopyranosidase) PpBGal42A from *Paenibacillus polymyxa* KF-1, which was successfully expressed in *Escherichia coli.* Using *p*NPG and *p*NPαArap as a substrate, PpBGal42A exhibited very good pH and temperature stability. PpBGal42A turned out to be a bifunctional β-galactosidase/α-Larabinopyranosidase with activity towards *p*NP-β-D-galactoside, *p*NP-α-L-arabinopyranoside, β-1,3/4/6-galactobiose, ginsenoside Rb2, AGP-I (β-1,3-galactan), potato galactan (β-1,4-galactan), and dGA (β-1,6-galactan). Furthermore, PpBGal42A hydrolyzed lactose and citrus pectin. Compared with other bifunctional enzymes (β-galactosidase/α-L-arabinopyranosidase) from various microorganisms, PpBGal42A is a superior candidate for advantageous application in polysaccharide degradation and the production of lactose-free milk.

## Figures and Tables

**Table 1:** Influence of different chemicals on the β-galactosidase activity of PpBGal42A.

Metal Ions or Chemicals	5 mM	50 mM
	Relative Activity (%)	Relative Activity (%)
None	100 ± 2.1	
NaCl	114.9 ± 6.7	90.2 ± 5.2
KCl	114.9 ± 5.4	83.0 ± 3.1
LiCl	108.8 ± 8.1	79.9 ± 9.2
CaCl_2_	110.2 ± 9.1	82.7 ± 3.5
MgCl_2_	87.7 ± 10.9	58.4 ± 5.8
BaCl_2_	90.2 ± 7.1	40.1 ± 4.6
MnCl_2_	-	-
FeSO_4_	9.8 ± 0.1	-
HgCl_2_	-	-
CuCl_2_	-	-
NiCl_2_	54.9 ± 3.8	-
ZnCl_2_	1.3 ± 2.2	-
EDTA	92.2 ± 8.9	68.3 ± 3.4
DTT	98.0 ± 10.1	74.4 ± 5.1

Results are presented as the mean ± standard deviation (*n* = 3).

**Table 2:** Determination of specific activities for recombinant PpBGal42A with nitrophenyl-linked substrates.

Substrate	Specific Activities (%)
*p*NP-β-D-galactopyranoside (*p*NPG)	100.0
*p*NP-α-D-galactopyranoside	<0.1
*p*NP-α-L-arabinopyranoside	56.7
*p*NP-α-L-arabinofuranoside	<0.1
*p*NP-α-D-glucopyranoside	<0.1
*p*NP-β-D-glucopyranoside	<0.1
*p*NP-α-D-mannopyranoside	<0.1
*p*NP-β-D-mannopyranoside	<0.1
*p*NP-α-D-xylopyranoside	<0.1
*p*NP-β-D-xylopyranoside	<0.1
*p*NP-α-L-fucopyranoside	<0.1
*p*NP-α-L-rhamnopyranoside	<0.1

**Table 3:** Degradation of pectin in combination with PpBGal42A and pectinase.

	Yield of Relaesing Sugar, μg
pectin + pectinase	579.1 ± 5.8
pectin + PpBGal42A	3.5 ± 0.3
pectin + pectinase + PpBGal42A	641.9 ± 6.2

**Table 4:** Comparison the enzymatic properties of PpBGal42A with other bifunctional enzyme (β-galactosidase/α-L-arabinopyranosidase).

Organism (Enzyme)	Family	Optimal Reaction Temperature (°C)	Thermal Stability Range (°C)		Optimal pH	Thermal Stability pH		Hydrolysis Property				Reference
			Time (min/h)	Residual Activity (%)		pH (min/h)	Residual Activity (%)	Activity for pNP	Activity for Disaccharides/Rb2	Activity for Polysaccharides	Hydrolysis of Lactose	
*Paenibacillus polymyxa* KF-1 (PpBGal42A)	42	30	20–30 (6 h)	90%	6.0	7–8 (12 h)	95%	*p*NPG, *p*NPA	β-1,3-galactobiose, β-1,4-galactobiose, β-1,6-galactobiose, Rb2	AGP-I, potato galactan, dGA	82% (24 h)	This study
*Bacillus* sp. KW1 (BaBgal42A)	42	45	30–45 (12 h)	75%	6.5	6.0–7.5 (12 h)	65%	*p*NPG, *o*NPG, *p*NPA, *p*NPF	NR	Galactan, arabinan, wheat arabinoxylan	80% (32 h)	[19]
*Bififidobacterium longum* H-1 (Apy-H1)	3	48	NR	NR	6.8	NR	NR	*p*NPG, *p*NPA	NR	NR	NR	[12]
*Clostridium cellulovorans* (BgaA)	42	30–40	50 (20 min)	0	6.0	6.0–8.0	NR	*p*NPG, *p*NPA, *p*NPF	NR	Arabinogalactan (larch wood)	NR	[34]
*Geobacillus* (Gan42B)	42	53	NR	NR	6.0	NR	NR	*p*NPG, *p*NPA, *p*NPF	β-1,4-galactobiose, Rb2	β-1,4-galacto-oligosaccharides	0	[35]

NR—not reported.

## Data Availability

Not applicable.

## Supplementary Materials

The following supporting information can be downloaded at: https://www.mdpi.com/article/10.3390/molecules28227464/s1, Figure S1: TLC analysis of ginsenoside conversion; Figure S2: Mw of PpBGal42A determined by SDS-PAGE; Table S1: Summary of expression and purification of recombinant PpBGal42A.
