# Supplementary material for: Cloning, Expression, Purification, and Characterization of a Novel β-Galactosidase/α-L-Arabinopyranosidase from Paenibacillus polymyxa KF-1
Source: Molecules. 2023 Nov 7;28(22):7464. doi: 10.3390/molecules28227464 (PMC10673005; doi:10.3390/molecules28227464)
Supplement: Supplementary file 1 [file molecules-28-07464-s001.zip › Figure S2.pdf]

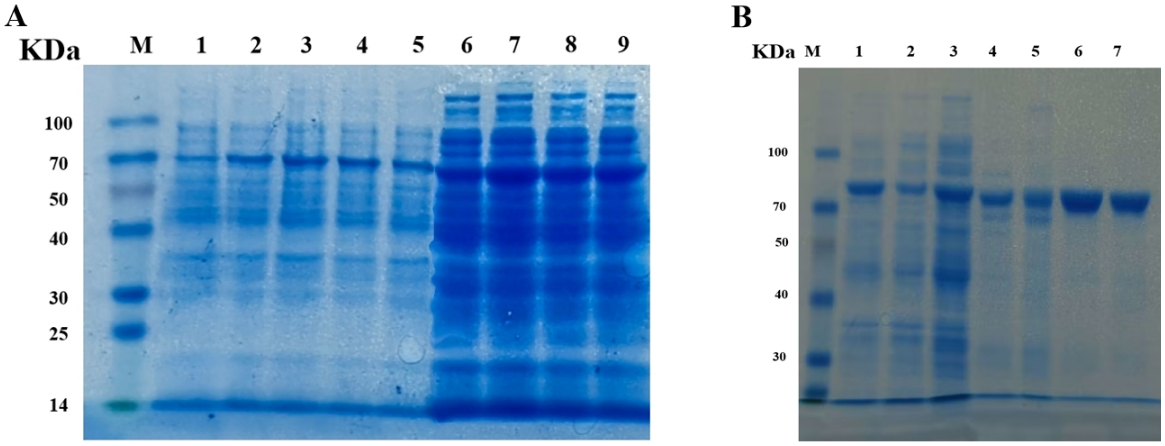

Figure S2. Mw of PpBGal42A determined by SDS-PAGE: M, Mw marker Blue Plus Protein Ladder, TransGen Biotech).

A.

(1) culture lysate before IPTG induction;

(2) 0.2 mmol concentration of IPTG after induction;

(3) 0.5 mmol concentration of IPTG after induction;

(4) 0.8 mmol concentration of IPTG after induction;

(5) 1.1 mmol concentration of IPTG after induction.

(6) Supernatant after crushing was induced by IPTG at 0.2 mmol concentration;

(7) Supernatant after crushing was induced by IPTG at 0.5 mmol concentration;

(8) Supernatant after crushing was induced by IPTG at 0.8 mmol concentration;

(9) Supernatant after crushing was induced by IPTG at 1.1 mmol concentration.

B. (1).culture lysate before IPTG induction;

(2) culture lysate after IPTG induction;

(3) Supernatant after crushing

(4) 5 mmol concentration of imidazole after elution of pure enzyme

(5) 10 mmol concentration of imidazole after elution of pure enzyme

(6) 20mmol concentration of imidazole after elution of pure enzyme

(7) 25 mmol concentration of imidazole after elution of pure enzyme
