# Supplementary material for: Cloning, Expression, Purification, and Characterization of a Novel β-Galactosidase/α-L-Arabinopyranosidase from Paenibacillus polymyxa KF-1
Source: Molecules. 2023 Nov 7;28(22):7464. doi: 10.3390/molecules28227464 (PMC10673005; doi:10.3390/molecules28227464)
Supplement: Supplementary file 1 [file molecules-28-07464-s001.zip › Table S1.pdf]

Table S1. Summary of expression and purification of recombinant PpBGal42A.

		IPTG concentration (mM)			
		0.2	0.5	0.8	1.1
**Crude Enzyme**	**mass(mg/L)**	45.9	63.0	58.2	60.3
**Pure Enzyme**	**mass(mg/L)**	7.8	9.1	5.3	6.2
