# Supplementary figures and images for: Cloning, Expression, Purification, and Characterization of a Novel β-Galactosidase/α-L-Arabinopyranosidase from Paenibacillus polymyxa KF-1
Source: Molecules. 2023 Nov 7;28(22):7464. doi: 10.3390/molecules28227464 (PMC10673005; doi:10.3390/molecules28227464)

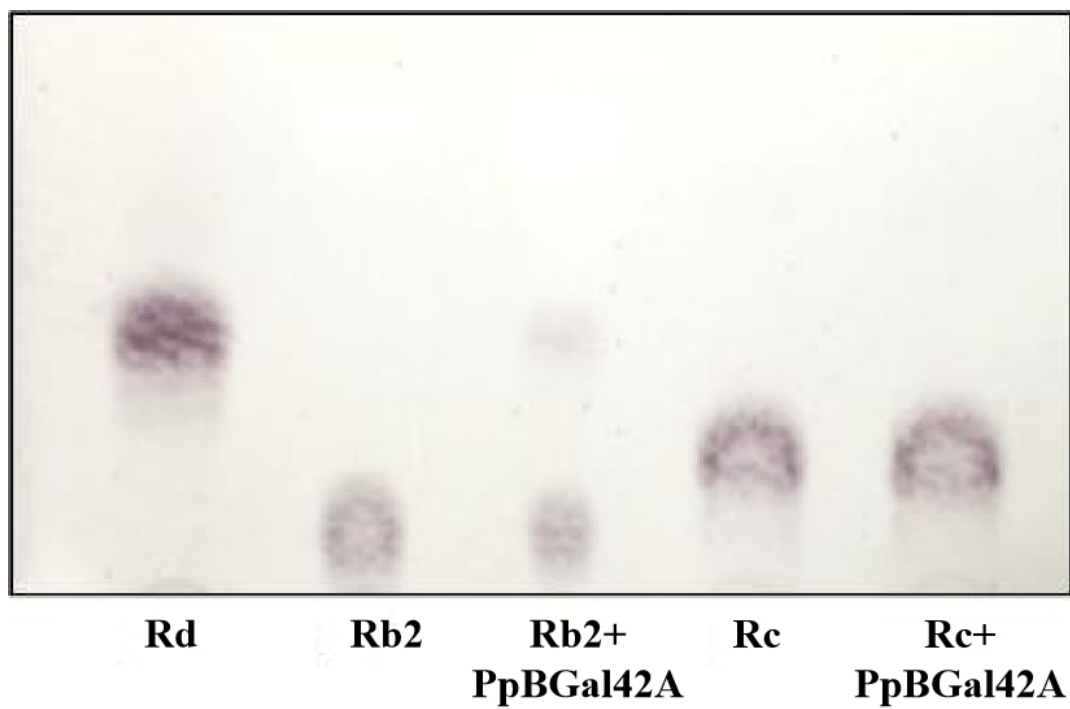

**Figure S1. TLC analysis of ginsenoside conversion.**

Supplement: Supplementary file 1 [file molecules-28-07464-s001.zip › Figure S1.pdf]
